# Vocal features obtained through automated methods in verbal fluency tasks can aid the identification of mixed episodes in bipolar disorder

**DOI:** 10.1038/s41398-021-01535-z

**Published:** 2021-08-02

**Authors:** Luisa Weiner, Andrea Guidi, Nadège Doignon-Camus, Anne Giersch, Gilles Bertschy, Nicola Vanello

**Affiliations:** 1 INSERM 1114, Strasbourg, France; 2 University Hospital of Strasbourg, Strasbourg, France; 3 Laboratoire de Psychologie des Cognitions, Université de Strasbourg, Strasbourg, France; 4 Dipartimento di Ingegneria dell’Informazione, University of Pisa, Via G. Caruso 16, 56122 Pisa, Italy; 5 Research Center “E. Piaggio”, University of Pisa, Largo L, Lazzarino 1, 56122 Pisa, Italy; 6 Fédération de Médecine Translationnelle de Strasbourg, Université de Strasbourg, Strasbourg, France

**Keywords:** Bipolar disorder, Diagnostic markers

## Abstract

There is a lack of consensus on the diagnostic thresholds that could improve the detection accuracy of bipolar mixed episodes in clinical settings. Some studies have shown that voice features could be reliable biomarkers of manic and depressive episodes compared to euthymic states, but none thus far have investigated whether they could aid the distinction between mixed and non-mixed acute bipolar episodes. Here we investigated whether vocal features acquired via verbal fluency tasks could accurately classify mixed states in bipolar disorder using machine learning methods. Fifty-six patients with bipolar disorder were recruited during an acute episode (19 hypomanic, 8 mixed hypomanic, 17 with mixed depression, 12 with depression). Nine different trials belonging to four conditions of verbal fluency tasks—letter, semantic, free word generation, and associational fluency—were administered. Spectral and prosodic features in three conditions were selected for the classification algorithm. Using the leave-one-subject-out (LOSO) strategy to train the classifier, we calculated the accuracy rate, the F1 score, and the Matthews correlation coefficient (MCC). For depression versus mixed depression, the accuracy and F1 scores were high, i.e., respectively 0.83 and 0.86, and the MCC was of 0.64. For hypomania versus mixed hypomania, accuracy and F1 scores were also high, i.e., 0.86 and 0.75, respectively, and the MCC was of 0.57. Given the high rates of correctly classified subjects, vocal features quickly acquired via verbal fluency tasks seem to be reliable biomarkers that could be easily implemented in clinical settings to improve diagnostic accuracy.

## Introduction

Mixed episodes, wherein depressive and manic symptoms co-occur, are frequently experienced during the course of bipolar disorder (BD), and are associated with a more recurrent and unfavorable illness course [1]. Among patients experiencing an acute mood episode, frequency of mixed depression and mixed mania range between 20–70% and 30–40%, respectively, depending on the samples, and the diagnostic thresholds used [1, 2]. The identification of mixed episodes has important consequences for care, as mixed episodes require different treatment options compared to their non-mixed forms. Indeed, patients with mixed mania have a significantly poorer response to lithium compared to patients with pure mania [3] but show better treatment responses with stabilizing antiepileptic drugs and some atypical anti-psychotics [3]. In individuals with mixed depression, antidepressants are less effective [4] and may increase the likelihood of a switch to mania, and risk of suicide [3, 4]. Therefore, antidepressant prescriptions need to be handled with caution, and the use of antipsychotics and mood-stabilizers has been warranted [4].

Prior studies have shown that clinicians often fail to recognize symptoms from the opposite polarity in predominantly depressive or manic episodes [5]. This might be due to an over-reliance on the more predominant symptoms presented by the patient, and to the specific phenomenology of mixed episodes, including increased anxiety and emotional lability related to hyperarousal [1, 6], which has been thus far overlooked by classifications [7]. Moreover, the restrictive diagnostic thresholds for symptoms of opposite polarity in mania and depression may also be involved in the underdiagnosis of mixed episodes [8]. In order to increase diagnostic specificity, in the most recent version of the *Diagnostic and Statistical Manual of Mental Disorders* (DSM-5) [9] symptoms shared by mania and depression (i.e., distractibility, irritability, and psychomotor agitation, or DIP) were excluded from the mixed symptoms specifier that can be applied to major depressive or hypomanic/manic episodes. However, DIP symptoms are commonly found during mixed episodes [7], and the DSM-5 criteria have been found to lack sensitivity, allowing the diagnosis of mixed depression for instance only in one out of four cases [8]. Recently, studies have shown that the presence of fewer concurrent symptoms from the opposite polarity, regardless of their overlapping nature—e.g., in the case of a depressive episode, a score above 2 on the Young Mania Rating Scale (YMRS) [10]—had better sensitivity and specificity than the current DSM-5 diagnostic criteria for mixed episodes “with mixed features” [2, 11].

There is a lack of consensus in the literature thus far on the diagnostic thresholds that could improve the detection accuracy of mixed episodes in BD. Here we investigated whether vocal features acquired via verbal fluency tasks (VFT) could accurately classify mixed states in BD using machine learning methods. To our knowledge, no large-scale studies have targeted the biomarkers that could aid the distinction between mixed and non-mixed BD episodes. Yet, recent studies have shown that automated methods relying on physiological parameters, e.g., speech prosody and heartbeat variability, could provide relevant information regarding mood states in BD [12, 13]. Voice acoustical analyses, in particular, may provide a powerful and easy to implement complement to the standard clinical interview for mood episodes in BD, as speech changes have been found to be sensitive and valid measures of depression and mania [12]. Specifically, through the automated use of speech parameters, promising results have been reported in the classification of depressive and manic episodes relative to euthymia [12, 14].

Very few studies have reported whether and how speech is modified during mixed episodes. Speech changes are characteristic of manic and depressive symptomatology, as assessed by specific items in clinician-rated scales such as the YMRS [10] (item 6 assesses speech rate and item 9 assesses loudness), and the Quick Inventory for Depressive Symptomatology (QIDS [15]; item 15 assesses speech rate), respectively. In depressive episodes, intonation has been typically described as flat, speech rate is slower than usual, and voice intensity and frequency are decreased [16]. Conversely, fewer studies have been conducted in mania, but faster than usual speech rate (i.e., pressure of speech), and increased voice volume are among the most commonly reported symptoms in mania [17], and have been hypothesized to be related to hyperarousal [14, 17]. In mixed episodes, a study from 1938 highlighted that, in one patient experiencing a mixed episode, pressure of speech was associated with vigorous articulatory movements, wide pitch range, fast speech tempo, and infrequent prosodic pauses [18].

From a psychoacoustic perspective, the correlates of such speech changes refer to mood-modulated prosodic and spectral features [14, 19]. According to Scherer’s [20] model, arousal, in particular, is associated with physiological changes in respiration, phonation, and articulation, which lead, in turn, to emotion and mood-specific patterns in acoustic parameters. For instance, speech fundamental frequency (F0), i.e., the inverse of the vocal folds opening and closing cycle period, which is related to the perceived pitch or voice tone, is used to voluntarily convey specific information to the listener, thus creating intonational events with language-specific meaning [14]. In addition to F0, long-term average spectrum (LTAS) has been described as a key feature involved in the differentiation of discrete emotions [21]. LTAS is related to voice quality, which is involved in how the listener perceives one’s voice as either creaky, breathy or tense [21]. Importantly, LTAS results from the speaker’s anatomy which determines both the width of the potential operating range, and its long-term muscular adjustments of the larynx or the supraglottic vocal tract [19]. Recently, Guidi et al. [14, 21] found that voice quality (LTAS) and prosodic features (F0 dynamics) were particularly sensitive and specific to longitudinally-evaluated non-mixed mood episodes in BD.

In this study, we investigated specific speech parameters—i.e., voice quality (LTAS) and prosody (F0 dynamics and pauses)—that have been found to be modulated by mood changes using VFT. VFT are easy to administer, well-validated and widely used neuropsychological tests targeting spontaneous word production during an allotted time [22]. In VFT, subjects are instructed to generate words according to specified rules based on phonemic or semantic criteria (“letter” and “semantic” fluency, respectively), in the absence of a specified criterion (free word generation), or through the continuous association of words following a cue word (associational fluency) [22]. The latter unrestrictive conditions are closer to natural discourse, while they also circumvent the methodological pitfalls of natural speech, such as pragmatics and syntax [23]. In BD, word count has been found to be impaired in letter and semantic VFT in euthymia [24]. Moreover, in unconstrained VFT (free word generation), Weiner et al. [23] found an increased number of switches from one conceptual unit (word or cluster) to another in patients with mixed symptoms compared to non-mixed depressed patients. VFT seems thus to be a sensitive means of measuring mixed symptoms found in depression and mania.

To our knowledge, studies focusing on whether and how vocal parameters could aid the identification of mixed episodes in BD are lacking. The present study aims at assessing the classification accuracy of mixed episodes, using a machine learning approach, via the automated analysis of voice quality (LTAS) and prosodic (F0 related features and pauses) vocal parameters obtained via different VFT conditions, i.e., differing in terms of their retrieval rules, but also, in associational tasks, in terms of the valence of the cue words [25]. Consistent with other studies which have used natural speech conditions for the classification of acute non-mixed states relative to euthymia [12], compared to a standard clinical assessment, we expected classification accuracy to be high in the detection of mixed relative to non-mixed episodes using vocal parameters in VFT.

## Methods

### Participants

Fifty-six patients ages 19–64 (M = 41.12, SD = 13.05) with BD were recruited from inpatient and outpatient clinics at the University Hospital of Strasbourg. Patients fulfilled the criteria for BD according to the DSM-5 [9]. Twenty-four patients had BD type 1, and thirty-two BD type 2. Patients with BD had no history of neurological disorder, ADHD, borderline personality disorder, or substance use disorder within the last 12 months. Mania and depression symptoms were assessed with the YMRS [10] and the QIDS-C16 [15]. Anxiety symptoms were assessed via the Beck Anxiety Inventory (BAI) [26]. Patients were considered to be in a predominantly depressive or manic/(hypo)manic episode if they fulfilled the DSM-5 criteria for either episode [9].

Given that mixed symptoms in depression might be observed with very few concurrent hypomanic symptoms [7, 11], our mixed depression group was defined based on the less restrictive criteria of Miller et al. [11]. In their study, mixed depression was operationalized as above threshold depressive symptoms (QIDS-C16 score >5), co-occurring with mild hypomanic symptoms (YMRS score >2 and <6). This cut-off has proved to be more sensitive to mixed depression than the DSM-5 criteria, as DIP features are not precluded from their criteria [11]. Moreover, we assessed anxiety via the BAI [26] because it has been consistently linked to mixed symptomatology in BD [1], and to speech changes in healthy individuals [20]. A YMRS score >5 was considered reflective of hypomania [27] and a QIDS-C16 score >5 was reflective of depression [15]. A mixed manic/hypomanic label was applied if manic and depressive symptoms were above the cut-off [28]. Consistent with Miller et al. [11] mixed depression was operationalized as above the threshold depressive symptoms (QIDS-C16 score >5), co-occurring with mild hypomanic symptoms (YMRS score >2 and <6). Nineteen patients were considered by clinicians as hypomanic and 8 had a mixed hypomanic episode. Among predominantly depressed patients, 17 were clinically assessed as mixed depressive and 12 had a pure depressive episode (cf. Table 1). Subjects provided written informed consent prior to inclusion in the study in accordance with the Declaration of Helsinki (ClinicalTrials.gov registration number: NCT02036606). This study was approved by the regional ethics committee of the East of France.

**Table 1:** Demographic characteristics of the patient samples.

	Hypomania	Mixed hypomania	Mixed Depression	Depression
	*n* = 19	*n* = 8	*n* = 17	*n* = 12
Age^a^	37.58 (13.52)	42 (10.3)	42.12 (12.75)	44.83 (14.43)
Sex (F/M)	12/7	4/4	14/3	8/4
YMRS^a^	12.58 (3.63)	9.25 (2.37)	4.37 (1.5)	0.83 (0.94)
QIDS-C16^a^	2.37 (1.5)	9.75 (3.01)	12.37 (3.95)	12.5 (3.58)
Lithium (% yes)	42%	12.5%	56%	50%
Anti-epileptics (% yes)	42%	50%	56%	50%
Antipsychotics (%yes)	63%	37.5%	37.5%	50%
Antidepressants (%yes)	26.5%	37.5%	37.5%	66.5%
Benzodiazepines (%yes)	15.5%	12.5%	25%	25%

*YMRS* Young Mania Rating Scale, *QIDS-C16* Quick Inventory of Depressive Symptomatology.

^a^Mean and standard deviation

### Materials and procedure

Following the aforementioned diagnostic clinical assessment, participants completed the Beck Anxiety Inventory (BAI) [26] prior to the administration of the VFT.

#### Verbal fluency tasks

Nine different trials belonging to four conditions of VFT were administered: (i) one free fluency trial, (ii) six associational fluency trials, (iii) one letter fluency trial, and (iv) one category fluency trial. The four conditions of the VFT were administered in a fixed order starting with the most unrestrictive condition [29]: first the free condition, followed by the six associational conditions, the letter condition, and the category condition. Participants’ oral production was recorded using the Audacity© software (44,100 Hz sampling frequency, 24-bit pulse code modulation). A microphone connected to a laptop was used. The microphone was kept approximately 60 cm far from the subjects’ mouth. The room was quiet with low reverberation levels.

#### Free fluency condition

In the free fluency trial, participants were asked to produce as many words as possible, with their eyes closed, during 150 s [30].

#### Associational fluency conditions

Subjects were orally presented with an initial cue word, and they were asked to produce words, during 120 s, with their eyes closed, following the presentation of the initial word. Prior to the task trials, subjects were provided with an example (i.e., the word “glass”). Two types of inductive words were used, i.e., concrete and abstract nouns. For each type, three disyllabic words were chosen, with a negative, neutral or positive emotional valence (for concrete words, “snake”, “kingdom”, and “swimming pool”, respectively; for abstract words, “pain”, “beginning”, and “courage”, respectively). Words within each triplet were matched in terms of their film subtitle-based frequency in French [31], their concreteness [32], and emotional valence ratings among French native speakers [33]. The six inductive words were presented in random order.

#### Letter fluency condition

Subjects were asked to produce as many words as possible starting with the letter ‘p’, with the exception of proper nouns, during 120 s [29].

#### Semantic fluency condition

Participants were asked to produce as many words as possible belonging to a specific category, i.e., “animals”, during 120 s [29].

### Extraction of vocal features

Speech features related both to prosodic information and voice quality were obtained. The analysis was based on a two-step procedure. First, single words were selected using a voice activity detection algorithm, then speech features were calculated for each word.

#### Word detection

The word detection algorithm analyzes the energy of the audio signal as well as its temporal and spectral features. Specifically, it consists of a modification of the signal intensity and zero crossing rate that checks whether the word candidate comprises voiced sounds, according to a spectral matching procedure based on the Camacho SWIPE’ algorithm [34]. This algorithm compares the spectral content of the audio signal with a spectral template of a sawtooth waveform, mimicking the glottal source signal characteristics. The resulting estimates consist of F0 (pitch) and the strength of the spectral matching (cf. Table 2). We then analyzed signal intensity, zero crossing rate, and spectral strength, to detect single words.

**Table 2:** Features used for the analysis of speech signals.

Feature name	Feature category	Definition	Meaning
MedianF_0_	Prosodic	Median of F_0_ values estimated within each word	Central tendency of voiced sound fundamental frequency
MadF_0_	Prosodic	Median Absolute Deviation of F_0_ values estimated within each word	Dispersion index of voiced sound fundamental frequency
*Amplitute*	prosodic	$\frac{{A_{rise} - \left| {A_{fall}} \right|}}{{A_{rise} + \left| {A_{fall}} \right|}}$	Relative size of F0 rising and falling phase amplitudes
*Duration*	Prosodic	$\frac{{D_{rise} - D_{fall}}}{{D_{rise} + D_{fall}}}$	Relative size of F0 rising and falling phase durations
Tilt^*^	Prosodic	$\frac{{Amplitude^ \ast + Duration^ \ast }}{2}$	Mean of Amplitude* and Duration* features
*PosSlope*	Prosodic	$\frac{{A_{rise}}}{{D_{rise}}}$	steepness of the F_0_ contour during rising phase
*AbsNegSlope*	Prosodic	$\frac{{\left| {A_{fall}} \right|}}{{D_{fall}}}$	steepness of the F_0_ contour during falling phase
*SumDer*	Prosodic	$\frac{{A_{rise}}}{{D_{rise}}} + \frac{{\left| {A_{fall}} \right|}}{{D_{fall}}}$	Sum of PosSlope and AbsNegSlope
*GlobalSlope*	Prosodic	$\frac{{A_{rise} - \left| {A_{fall}} \right|}}{{D_{rise} + D_{fall}}}$	F_0_ slope between the first and the final F_0_ values in each voiced segment
Mean_Pause	Prosodic	The mean across a VFT of pause lengths between two consecutive words	Position index of pause length distribution
Std_Pause	Prosodic	The standard deviation across a VFT of pause lengths between two consecutive words	Dispersion index of pause length distribution
Mean_Speech	Prosodic	The mean word length across a VFT	Position index of word length distribution
Std_Speech	Prosodic	The standard deviation of word length across a VFT	Dispersion index of word length distribution
LTAS_F_median	Voice quality	the median frequency of a power spectrum divides the total power in two halves	LTAS shape feature. Central tendency index of the LTAS spectrum distribution. Relative Contribution of high and low frequencies
LTAS_A_median	Voice quality	The amplitude of the LTAS spectrum corresponding to LTAS_F_median	
LTAS_Max_A	Voice quality	The maximum amplitude of LTAS spectrum	
LTAS_Max_A_F	Voice quality	The frequency values corresponding to LTAS_Max_A	LTAS shape feature. Its value is expected to be lower than LTAS_F_median
LTAS_slope	Voice quality	$\frac{{{\rm{LTAS}}\_{\rm{Max}}\_{\rm{A}} - {\rm{LTAS}}\_{\rm{A}}\_{\rm{median}}}}{{{\rm{LTAS}}\_{\rm{Max}}\_{\rm{A}}\_{\rm{F}} - {\rm{LTAS}}\_{\rm{F}}\_{\rm{median}}}}$	LTAS shape feature: it is related to the slope of the LTAS spectrum between the peak and the amplitude corresponding to median frequency. The lower (negative), the smaller the contribution of higher frequencies
LTAS_Ratio_Max	Voice quality	$\frac{{{\rm{LTAS}}\_{\rm{Max}}\_{\rm{A}}}}{{{\rm{LTAS}}\_{\rm{Max}}\_{\rm{A}}\_{\rm{F}}}}$	LTAS shape feature: it is related to the slope of the LTAS spectrum between the origin and the LTAS peak. Given that the maximum peak is at low frequencies, it weights amplitude of lower frequencies
LTAS_Ratio_Median	Voice quality	$\frac{{{\rm{LTAS}}\_{\rm{A}}\_{\rm{median}}}}{{{\rm{LTAS}}\_{\rm{F}}\_{\rm{median}}}}$	LTAS shape feature: it is related to the slope of the LTAS spectrum between the origin and the value corresponding to the median frequency.

#### Speech feature estimation

We estimated specific features related to prosody (F0 related features and pauses) and voice quality (LTAS-related features; see Table 2).

**Fig. 1:**
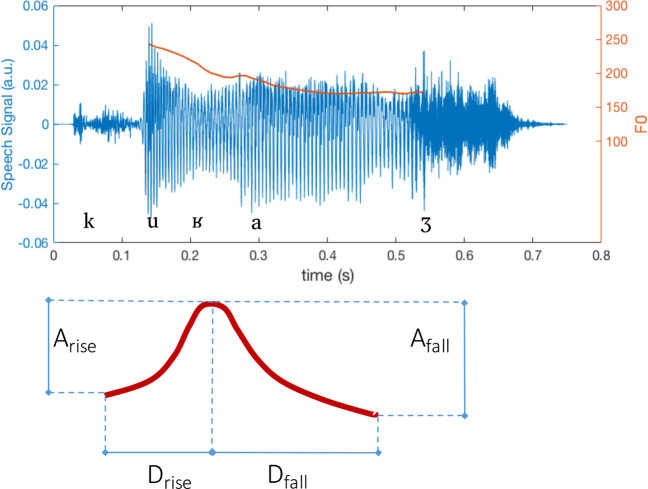
F0 dynamics of the word "courage" and Taylor’s (2000) tilt model. Upper. The time course of audio signal related to the French word “courage” along with its phonetic transcription. Fricative voiceless sounds are characterized by more rapid changes with respect to voiced sound (central part of the word). For voiced sounds, the fundamental frequency (F_0_) can be estimated and its time contour is shown in red. Lower. Taylor’s (2000) tilt model, whereby the falling phase is present thus resulting in geometric parameters as Duration*, and Amplitude* equal to −1.

Prosodic features were obtained after word segmentation, by calculating pauses between words as well as word length, and by estimating F0 dynamics (Fig. 1). F0 dynamics (temporal windows of 10 milliseconds) were obtained using the SWIPE’ algorithm. For each word, summary statistics of F0, e.g., median and median absolute deviation, were estimated. Nine features describing the F0 contour for each word were also analyzed. These features correspond to Taylor’s [35] tilt intonational model and describe the relative size and duration of intonational events (Fig. 1).

We extended the use of these features to all the voiced segments within each word [14]. The resulting features, Amplitude*, Duration* and Tilt*, are described in Table 2. These features refer to the shape of the F0 contour within each voiced segment, which allow for the analysis of F0 dynamics, i.e., both rising and falling phases within each voiced segment [14] (Table 2).

**Fig. 2:**
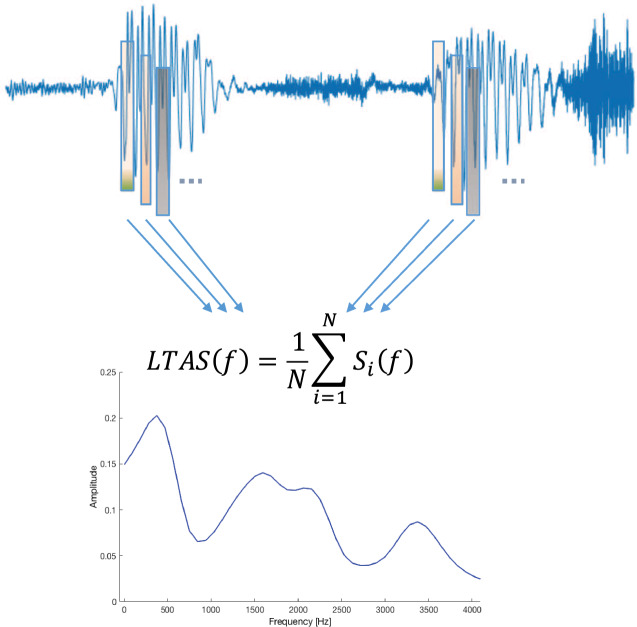
Long-term average spectrum (LTAS) estimation strategy and example. Upper. Long-term average spectrum estimation strategy. Lower. An example of LTAS. The features, provided in Table 2, were identified to parsimoniously describe the LTAS shape.

Voice quality features were obtained by estimating the LTAS using F0-correction [36]. LTAS is used to identify long-term muscular settings of the larynx and the vocal tract, that deviate from neutral settings [19]. Given that LTAS is estimated using a moving time window approach applied to the voice signal, the obtained amplitude spectra are averaged over all windows. F0 correction reduces the influence of F0 on spectral characteristics, such as the articulatory movements involved in LTAS. Using this procedure, we aimed at minimizing the overlap between F0- and LTAS-related speech features (see Fig. 2).

To estimate F0-corrected LTAS, we modulated the size and the location of the moving window in order to analyze a single glottal cycle for each window using the DYPSA algorithm [37]. The LTAS was estimated using a frequency resolution of 150 Hz, within the whole frequency range (i.e., 0–2,2050 Hz). The selected LTAS features are described in Table 2.

### Statistical analyses

Statistical analyses were defined in advance and were performed by researchers who were blind to the diagnostic status of patients. In order to select the most relevant vocal parameters for the classification algorithm, we calculated Spearman rho coefficients between clinical questionnaire scores—i.e., YMRS total, QIDS-C16, and BAI—and vocal measures obtained in the nine VFT within two sample of patients—manic and mixed manic, and depressed and mixed depressed. The VFT and the vocal features that were correlated to the clinical measures were then entered in our classification algorithm.

A support vector machine (SVM) classifier [38] was used to train discriminative models using the extracted measures. The task consisted of a binary classification, where a model was trained to discriminate between two distinct acute episodes, i.e., manic versus mixed manic and depression versus mixed depression. The leave-one-subject-out (LOSO) strategy was used to train the classifier: LOSO consists of removing all the observations related to a subject (validation set) and train the classifier on the remaining observations (training set). The subject is then classified, and the predicted label is compared with the clinical scoring. This operation is repeated for the remaining subjects in the dataset until a predicted label is obtained for each subject. Before the training and the test steps, each speech feature underwent a subject-specific normalization step, to remove subject-specific speech characteristics, such as gender or vocal tract signature. The MadF0, i.e. the median absolute deviation of F0, obtained for each task was normalized through the use of the median value of F0. This was used to compensate for higher values of F0 variability at higher median values of F0 (measured in Hz) due to intra-individual speaker characteristics. The remaining features were normalized using a z-score normalization, i.e., subtracting the mean and dividing by the standard deviation estimated across each of the 6 tasks.

To account for the potential effect of drugs on classification results, we calculated the chlorpromazine equivalent doses of the antipsychotic drugs, as antipsychotics have been found to have a significant effect on speech [39], and they are the first-line treatment for mixed episodes in BD [4]. Chlorpromazine equivalent dose was hence adopted as descriptor, and as an input to the classifiers along with the speech features.

Classical measures of classification performance were estimated, and the confusion matrix was reported. Specifically, positive and negative predictive values, indicated as PPV and NPV respectively, as well as sensitivity and specificity were estimated. Moreover, we also considered three complementary measures of classification: i.e., accuracy, F1 score, and Matthews correlation coefficient (MCC). Accuracy is defined as the sum of true positive and true negative results divided by the total positive and negative results. F1 score considers both the precision and the recall of the test to compute the score: precision refers to the number of correct positive results divided by the total number of positive results returned by the classifier, and recall is the number of correct positive results divided by the number of samples that should have been identified as positive. The different measures were estimated using mixed symptoms as a target (i.e., positive state). The confusion matrix describes the number of classified and misclassified subjects in both categories. The confusion matrix thus allows to have a quick and detailed view of the classification performances. The Matthews correlation coefficient (MCC) was used as a compact descriptor of the confusion matrix results, measuring the relationship between the observed and the ideal results. MCC is not biased by the number of observations nor does it depend on the choice of the target (i.e., positive state) [40]. MCC higher than 0.5 was expected, reflecting a strong positive relationship between the actual and the ideal classification results. MCC offers a robust measure of classification performance and can complete the information gained by the accuracy and the F1 score. In fact, it can be used in the case of unbalanced data, that is, when the number of subjects in the two groups are different, whereas accuracy can be biased by the results in the group with more subjects [40]. Moreover, MCC considers the overall performances related to the classification in both groups, while F1 is related to precision and recall and does not consider true negatives.

## Results

### Demographic and clinical data

Four patients were not taking any psychotropic medication at the time of the assessment. Of the remaining 52 patients, 45.3% were taking lithium, 53.9% were prescribed antiepileptic drugs, 61.5% were taking antipsychotics, 44.2% were on antidepressants, and 23.1% were taking benzodiazepines. Detailed demographic data are presented in Table 1.

### Speech feature and task selection

In the mixed manic and manic groups (*n* = 27), higher depression scores, measured by the QIDS-c16, were correlated to elevated voice quality parameters (LTAS_A_median, LTAS_Max_A_F, and LTAS_Ratio_Median) on the ‘snake’ condition only. In the mixed depression and depression groups (n=29), higher YMRS total score was correlated to higher median F_0_, higher F0 variability as expressed by MadF_0_ (the dispersion of voiced sound fundamental frequency) and higher Tilt* (mean of amplitude and duration) feature on the semantic, the letter, as well as on several conditions of the associational VFT. Given these results, the selected features for the mixed versus non-mixed algorithm were respectively for predominantly (i) manic and (ii) depression groups: (i) LTAS measures obtained in the “snake” associational VFT condition, and (ii) MedianF_0_, MadF_0_, Duration*, in the semantic and “beginning” associational VFT conditions.

### Classification results

Among the predominantly depressed patients, mixed depression was correctly classified in 15 cases, and 2 patients were misclassified as depressed. Pure depression, on the other hand, was correctly classified in 9 cases, and misclassified in 3 of them. The resulting accuracy and F1 scores were very high, i.e., respectively 0.83 and 0.86, and the MCC was equal to 0.64, revealing a good classification performance. Correctly classified mixed depression cases had, on average, higher anxiety scores, as measured by the BAI, compared to misclassified cases of mixed depression. One misclassified case had a predominantly irritable clinical presentation (YMRS score on the irritability item of 2), whereas the other was more agitated (YMRS score on the agitation item of 2). Classification and descriptive results on clinical measures are presented in Tables 3 and 4.

**Table 3:** Classification results in depression groups and descriptive clinical measures (mean and SD).

	Classification	*n*	YMRS	QIDS-C16	BAI
Depression	Correct	9	0.78 (0.97)	12.67 (3.77)	18.33 (6.12)
Depression	Incorrect	3	1 (1)	12 (3.60)	16.67 (13.05)
Mixed depression	Correct	15	4.43 (1.60)	12.14 (4.11)	29.14 (11.75)
Mixed depression	Incorrect	2	4 (0)	14 (2.83)	16.50 (13.43)

*YMRS* Young Mania Rating Scale, *QIDS-C16* Quick Inventory for Depressive Symptomatology, *BAI* Beck Anxiety Inventory.

**Table 4:** Classifier performance measures for depression groups (mixed symptoms as target).

NPV	PPV	Spec	Sens	Acc	F1	MCC
0.82	0.83	0.75	0.88	0.83	0.86	0.64

*NPV* negative predictive value, *PPV* positive predictive value, *Spec* specificity, *Sens* sensitivity, *Acc* accuracy, *F1* F1 score, *MCC* Matthew’s correlation coefficient.

Among predominantly hypomanic patients, mixed mania was correctly classified in 6 cases, and misclassified in 2. Pure manic/hypomanic states were correctly classified in 17 cases and misclassified in 2. The accuracy and F1 scores were high, i.e., 0.86 and 0.75, respectively. The good performance of the classifier, as applied to the unbalanced dataset, was confirmed by the MCC whose result was 0.57. Correctly classified mixed manic/hypomanic, but also pure manic/hypomanic states, had, on average, higher anxiety scores on the BAI, compared to misclassified cases. Dysphoric psychotic features were part of the ongoing episode in the two misclassified mixed manic cases. Classification and descriptive results on the clinical measures are presented in Tables 5 and 6.

**Table 5:** Classification results in manic groups and descriptive clinical measures (mean and SD).

	Classification	*n*	YMRS	QIDS-C16	BAI
Mania	Correct	17	12.82 (3.56)	2.47 (1.54)	14.94 (9.84)
Mania	Incorrect	2	10.50 (4.95)	1.50 (0.71)	2 (2.82)
Mixed mania	Correct	6	9.50 (2.74)	9.33 (3.44)	22.20 (10.42)
Mixed mania	Incorrect	2	8.50 (0.71)	11 (0)	18.5 (12.01)

*YMRS* Young Mania Rating Scale, *QIDS-C16* Quick Inventory for Depressive Symptoms-Clinician version, *BAI* Beck Anxiety Inventory.

**Table 6:** Classifier performance measures for manic groups (mixed symptoms as target).

NPV	PPV	Spec	Sens	Acc	F1	MCC
0.89	0.75	0.89	0.75	0.86	0.75	0.57

*NPV* negative predictive value, *PPV* positive predictive value, *Spec* Specificity, *Sens* sensitivity, *Ac* Accuracy, *F1* F1 score, *MCC* Matthew’s correlation coefficient.

The analyses were repeated using information about antipsychotic medication (chlorpromazine equivalent dose) as a predictor. The classification results did not improve when medication was added to the input features of the classifier. As a matter of fact, the best classification performances were not obtained when any of the selected speech features was substituted by medication.

## Discussion

Our classification results suggest that vocal features obtained through automated methods in VFT are sensitive measures of manic and depressive symptoms in BD, even in their milder forms, such as subthreshold hypomanic symptoms concurrent with a depressive episode (i.e., mixed depression). Strikingly, ours is the first study to show that classification accuracy using vocal acoustic measures is not only high in acute relative to euthymic states [12, 14, 21], but also in mixed relative to non-mixed acute episodes of the same polarity. These results bear important implications for clinicians, given the high rates of misdiagnoses of mixed states in clinical settings [1], and the higher risk of suicide associated with mixed episodes [1, 3].

Our accuracy results are higher than those previously reported in the classification of hypomanic or depressive episodes relative to euthymia based on natural speech conditions obtained via smartphone [12, 41]. Such is particularly the case for the classification of depression relative to euthymia, whose accuracy results were found to be lower (i.e., 0.68) than those reported for hypomanic states (i.e., 0.74) in the largest study conducted to date, with 28 patients with BD [12]. In our study which had the largest sample size to date (*n* = 56), classification performances were very high for both mixed depression and mixed mania, suggesting that automated methods relying on specific prosodic and spectral features can correctly classify most cases of patients presenting with mixed symptoms. Specifically, in the hypomanic groups, where the number of subjects differed considerably, it is important to highlight the good classification performance found with the MCC, which, unlike accuracy, is unbiased by unequal sample sizes.

These results may seem surprising, inasmuch as classification accuracy could be expected to be higher when identifying the presence relative to the absence of symptoms (i.e., acute versus euthymic states), rather than the presence of subthreshold manic and depressive symptoms in acute episodes of opposite polarity. The selection of vocal parameters used as classifiers, the specific phenomenology of mixed states, but also the manner through which voice samples were acquired might explain these results.

Firstly, in our study, we focused on a relatively small set of vocal features which had been consistently linked to emotion and mood-modulated speech changes in healthy individuals [20], and in some studies conducted in individuals with BD [14, 21]. Other studies have used automatic systems to produce a high dimensional description of the voice signal, but they did not specify which features contributed to the classification results [12, 41]. Hence, the underlying vocal and clinical mechanisms involved in the results could not be fully interpreted.

LTAS (voice quality), for instance, has been suggested to highlight different vocal tract settings across different mood states [21]. In our study, subjects with depressive symptoms concurrent with hypomania (i.e., mixed hypomania) showed larger amplitude values of low frequency formants as well as a flatter spectrum. This pattern of results differs from some reports in unipolar depression whereby a decrease of second and third formant was found with increasing depression severity [42, 43]. However, consistent with our results, in a longitudinal study in BD [21], larger amplitude of high frequency components was found in depression compared to euthymia. Hence, it is possible that LTAS features in bipolar depression differ from those found in unipolar depression, on the one hand, and that depressive symptoms concurrent with hypomania (i.e., mixed hypomania) are characterized by a flatter spectrum akin to bipolar depression [43].

In predominantly depressive episodes, concurrent hypomanic symptoms were associated with more variable intonation, as reflected by larger values of tilt features, such as Duration*. Given that the opposite pattern of results—i.e., lower median pitch and flat intonation—has been reported in patients with non-mixed depression [16, 20], our results are the first to show that voice acoustic measures are different in mixed depression compared to pure depression [18]. Specifically, more dynamic intonation characteristics (translated by tilt) were found in mixed depression compared to pure depression [18]. Given that increased self-rated anxiety was significantly correlated to higher fundamental frequency (F0; pitch) values, it is likely that increased arousal in mixed depression is involved in the more dynamic intonation found in mixed relative to non-mixed depression [1].

In addition to heightened anxiety, different clinical symptoms might be related to speech peculiarities in mixed episodes. It should be noted that, in our sample, anxiety scores were higher in mixed relative to non-mixed episodes, but also in correctly classified versus misclassified mixed cases. This suggests that increased anxiety might be at least partially involved in the vocal changes that are determinant for the classification of mixed relative to non-mixed patients [1, 44]. These findings are consistent with those from a number of studies showing increased fundamental frequency (F0) (i.e., higher pitch), in patients with anxiety disorders [44], and in healthy anxious individuals [20]. However, the phenomenology of mixed episodes is polymorphous, and might also encompass non-anxious forms. Heterogeneous clinical presentations among patients with mixed episodes might thus explain some of our results. Indeed, according to a study using principal component analysis by Perugi et al. [45], anxiety is a clinical dimension found in most, but not all, subtypes of severe mixed episodes in BD; these include, for instance, predominantly agitated-irritable mixed depression, anxious-irritable-psychotic mania, and retarded-psychotic mixed depression. In healthy individuals, Laukka et al. [46] found that irritability and resignation were associated with specific changes in F0 (pitch), voice quality (LTAS), and voice intensity measures. Given that our misclassified mixed patients were less anxious but had more psychotic features in the two mixed manic patients, and were more irritable and agitated, for the two misclassified mixed depression cases, it is possible that these clinical dimensions are involved in our results. Hence it seems particularly important to increase our understanding of the specific phenomenology of mixed symptoms in order to improve diagnostic accuracy in clinical settings [7, 11]. In our study, instead of applying the DSM-5 criteria [9], we used a data-driven approach based on Miller et al. [11] and Suppes et al. [28] to determine whether patients were in a mixed episode or not. This approach has been favored in a number of recent studies which argue for the use of a less-restrictive diagnostic algorithm for the diagnosis of mixed states, including overlapping symptoms (distractibility, irritability, and psychomotor agitation) [7, 11, 45, 47]. While the algorithms we used were less restrictive than the ones proposed by the DSM-5, there is an ongoing debate whether even less restrictive approaches focusing on specific features should be favored [7]. It is noteworthy that incorrectly classified pure depression individuals (as mixed depression) had a very low YMRS (hypomanic) score of 1. In a recent study [47], a YMRS score of 1 in subjects with bipolar depression was associated with increased racing thoughts, and a mixed-suggestive clinical picture characterized by hyperarousal. Hence, it is possible that misclassified subjects with ‘pure depression’, who had a YMRS score of only 1, might in fact have a mixed-suggestive clinical presentation, including mild vocal changes that were captured here via automated methods.

Another aspect that might have contributed to our results is the task we used. Unlike studies using natural speech recorded during phone calls [12, 41], which relied on long and diverse speech samples, our vocal measures were obtained through single verbal fluency trials. Most studies conducted in people with BD have focused on the use of free speech samples (i.e., recorded conversations) [12, 41]. This procedure has the advantage of being more ecologically valid than other widely used procedures (e.g., reading), as speech is captured either during a monologue (i.e., describing a memory) or a dialogue (i.e., social interaction) [48]. However, it also has disadvantages, as it requires the acquisition of a large amount of data (e.g., hundreds of hours of recorded conversations) due to the high contextual dependence of the data (e.g., kind of interaction or text read, and type of device used) [49]. Moreover, free speech often relies on the recording of personal conversations, and this might raise more ethical concerns than the use of standardized cognitive tasks [48]. Both issues might limit the use of these tasks in clinical settings. Conversely, VFT are an economic and standardized means for measuring language and speech production which circumvent some of the pragmatic and syntax confounders inherent to free speech [22]. Importantly, VFT have been widely used in BD, and have been found to tackle the semantic abnormalities that characterize manic speech [23, 24]. Moreover, a growing body of evidence suggests that there are moderate-to-strong correlations between laboratory-based cognitive performances, such as those acquired here via VFT, and performances acquired in natural settings [50].

In our study, three VFT conditions, lasting 120 s each, were selected as classifiers—i.e., two associational conditions and the semantic condition of VFT. Associational and semantic conditions of VFT tackle how words stored in semantic memory are retrieved in a relatively spontaneous fashion [25]. Word count in the semantic VFT has been found to be disproportionately impaired in BD [24], which has been linked to functional semantic abnormalities [23]. Anomalous word retrieval based on semantic cues might thus have interacted with some of the speech changes relevant for mood state classification (i.e., interjections, intonation, and voice quality changes). However, given that speech rate and pause duration (i.e., a proxy of word count) were not among the features correlated with depressive and manic symptoms in our study, it seems unlikely that semantic abnormalities alone could subtend the vocal changes that allowed the classification of mixed versus non-mixed episodes. Consistent with one previous study that found that positive affective category cues were related to greater number of words in VFT in euthymia relative to healthy controls [25], we found here that emotion category cues had an impact on the voice quality features (LTAS) of word production in BD. Indeed, in the manic/hypomanic groups, higher depression scores were correlated to voice quality (LTAS) features on the negative emotion category only (‘snake’ condition), highlighting a possible interaction between mood-discrepant emotion and word output in individuals with manic symptoms.

There are some limitations to our study. First, given the small number of misclassified patients, our interpretations are based on a small sample of patients. The limited amount of data also affects the generalizability of our classifier: in fact, we could not split the data in a training set, validation set, and test set. However, this issue was alleviated by the leave one subject out (LOSO) cross validation procedure that we used, which provides a good estimate of classifier performance [51]. Cross-sectional studies with larger samples are thus needed to further investigate the relationship between specific mood dimensions and classification results in BD. Second, the effect of medication on voice parameters in BD is still largely unknown, although antidepressant medication has been linked to greater pitch variability and improved speech tempo in unipolar depression [16]. Further studies assessing this particular domain are needed. Third, the predictive value of acoustic measures in detecting mixed symptoms in clinical contexts is still unknown but should be valuable for the follow-up of patients and the assessment of treatment response and risk of suicide [52, 53]. This is particularly relevant in the context of mixed episodes, as they are associated with an increased risk of suicide [1, 3]. Studies with a longitudinal design are hence warranted to address this point. Relatedly, thus far, studies in BD focused on voice data acquired over longer time periods, hence allowing to model intra-subject changes. This has proved useful to track mood changes (euthymia relative to depression or mania) [12, 14, 21], to build personalized models, and to investigate the long-term development of the illness [54, 55]. Studies with a longitudinal design are needed in order to address the question of whether speech samples acquired via VFT over several time points can aid the classification of mood states in BD, including mixed episodes. Fourth, VFT speech features were acquired in a laboratory setting, and the feasibility and the utility of this task in tracking mood changes in natural settings remain to be tested [48].

In sum, we found high rates of correctly classified subjects based on prosodic and spectral features obtained in three conditions of VFT. Our results suggest that VFT can be a valid and economic means of acquiring speech samples in patients with BD. More specifically, voice quality, pitch, and intonation measures acquired via VFT appear to be reliable and informative potential biomarkers regarding mixed symptoms in acute episodes of BD. Studies should consider investigating the additive value of combining semantic and speech measures in VFT to the classification of acute mood states in BD. Since most mixed cases are undiagnosed in clinical settings [1, 5], and are associated with an increased risk of suicide [1, 3], vocal features quickly acquired via VFT have the potential to complement the clinical assessment of patients presenting with a mood episode and improve the clinical management of mixed states.

## Data Availability

The data that support the findings of this study are available from the corresponding author upon request.
